# How can we balance risk and benefit of interleukin-18 armored T cell therapies?

**DOI:** 10.3389/ebm.2026.10938

**Published:** 2026-02-06

**Authors:** Paulina Vicenova, John Maher

**Affiliations:** 1 CAR Mechanics Lab, King’s College London, School of Cancer and Pharmaceutical Sciences, London, United Kingdom; 2 Leucid Bio Ltd., Guy’s Hospital, London, United Kingdom; 3 Department of Immunology, Eastbourne Hospital, Eastbourne, East Sussex, United Kingdom

**Keywords:** chimeric antigen receptor (CAR) T cell, cytokine release syndrome (CRS), IL-18 binding protein (IL-18BP), interferon-γ (IFN-γ), interleukin-18 (IL-18), T cell redirected for universal cytokine-mediated killing (TRUCK), TCR-engineered T cell, tumor microenvironment (TME)

## Abstract

CD19-specific CAR T cells engineered to secrete a constitutively active form of the pro-inflammatory cytokine, interleukin (IL)-18 have demonstrated impressive efficacy in a recent clinical trial involving subjects who had failed prior CAR T cell therapy. Corroborating these clinical data, preclinical studies of IL-18-armored CAR and T cell receptor-engineered T cells have demonstrated enhanced anti-tumor activity in several xenograft and syngeneic mouse cancer models. Interleukin-18 improves tumor clearance via direct effects on CAR T cells and indirect actions on cells on a variety of host immune cells, including natural killer, macrophage and dendritic cells. Compared to unarmored CAR T cells, IL-18-secreting CAR T cells are less exhausted, expand more efficiently and produce greater quantities of interferon (IFN)-γ. However, upregulated circulating IL-18 and its downstream mediator, IFN-γ, are also associated with systemic toxicities which have proven to be severe on occasions. In light of this, several groups have developed strategies that set out to restrict IL-18 release or biological activity to the tumor microenvironment. Among these, CAR T cells armored with NFAT-inducible IL-18 are now undergoing clinical testing. The evaluation of inducible or tumor-selective IL-18 deployment will show whether it is possible to minimize IL-18 related systemic toxicities while preserving localized amplification of anti-tumor activity.

## Impact statement

Although successful in the treatment of specific blood cancers, CAR T cell therapy has shown limited efficacy against solid tumors. A key barrier in this regard is the highly immunosuppressive nature of the solid tumor microenvironment (TME). One proposed means to address this entails the co-engineering of CAR T cells to produce interleukin (IL)-18, an approach that is currently being investigated in clinical trials. This minireview provides an overview of published clinical and preclinical studies of IL-18 armoring. We conclude that IL-18 consistently improves the anti-tumor efficacy of CAR T cells, but may elicit toxicities that arise from its pro-inflammatory properties. We also describe a number of strategies that set out to harness this cytokine in a more tumor-targeted and ultimately safer manner.

## Introduction

Chimeric antigen receptors (CARs) are synthetic transmembrane proteins that redirect the MHC-independent activation of immune effector cells (notably T cells) when they encounter native cell surface antigens [1]. As of December 2025, the United States Food and Drug Administration had approved seven autologous CAR T cell therapies targeting either CD19 in B-cell malignancies or B cell maturation antigen in multiple myeloma [2–4]. Recent years have seen an increase in global efforts to extend CAR T cell therapy to solid tumors, with over 300 registered clinical trials collectively targeting 46 distinct solid tumor-associated antigens [5].

To achieve clinical efficacy, particularly against solid cancers, CAR T cells need to avoid attenuation by immunosuppressive cells residing in the tumor microenvironment (TME) [6]. Regulatory T cells (Tregs), myeloid-derived suppressor cells (MDSCs) and M2 polarized macrophages can all inhibit T cells through inhibitory immune checkpoints or immunosuppressive factors, such as transforming growth factor-beta (TGF-β) and interleukin (IL-)10 [7].

Fourth generation CAR T cells, also known as armored CARs or TRUCKs (T cells Redirected for Universal Cytokine Killing), are designed to counteract the immunosuppressive TME by secreting pro-inflammatory cytokines [8, 9]. Commonly used examples include IL-7, IL-12, IL-15 or IL-18 [10–13]. In this minireview, we focus specifically on IL-18 and on strategies to maximize the therapeutic index of this approach.

Historically, IL-18 was known as interferon (IFN)-γ inducing factor owing to its ability to enhance IFN-γ secretion by CD4^+^ T cells, CD8^+^ T cells and Natural Killer (NK) cells [14–16]. Interleukin 18 is produced mainly by macrophages and dendritic cells (DCs) and is released as an inert precursor known as pro-IL-18. Canonical activation of pro-IL-18 is mediated by caspase-1 cleavage in the inflammasome, after which biologically active IL-18 is secreted through Gasdermin-D plasma membrane pores [17–19]. Upon release, IL-18 either binds to IL-18 receptor (IL-18R)α on the surface of T cells and NK cells or is neutralized by a soluble decoy receptor, IL-18 binding-protein (IL-18BP) [20, 21]. The interaction between IL-18Rα and IL-18 is stabilized by the accessory receptor IL-18Rβ, which facilitates activation of the transcription factor, NF-κB through the adaptor proteins, TRAM (TRIF-related adaptor molecule) and MyD88 (myeloid differentiation primary response protein 88) [22, 23].

Interleukin 18 exerts pleiotropic proinflammatory actions that include NK cell activation, DC maturation and context-dependent activation of either Th1 or Th2 responses [24–26]. The anti-tumor effects of IL-18 alone or in combination with IL-12 were reported in mouse models as early as 1998 [27–29]. Subsequent clinical trials of recombinant human IL-18 confirmed that it had a reasonable safety profile (albeit linked to some grade 3 and one grade 4 adverse reactions) [30, 31]. However, anti-tumor efficacy was insufficient [30, 31], prompting research into IL-18-based combination therapies including CAR T cell approaches.

## IL-18 armoring improves anti-tumor activity of engineered T cells in pre-clinical models

Armoring/supplementation of either CAR- and T cell receptor (TCR)-engineered T cells with IL-18 has been evaluated in several independent preclinical studies, employing both xenograft and syngeneic mouse models (Table 1). In all but two cases, IL-18 armoring was achieved by stable viral transduction. By contrast, Huang et al. [40] administered mature IL-18 protein by intraperitoneal injection, while Olivera et al. [43] transiently electroporated T cells with IL-18 mRNA, IL-12 mRNA, or both. In 13 of these studies, IL-18 was shown to boost anti-tumor efficacy. Exemplifying this, Chmielewski et al. [37] and Ng et al. [36] both showed that IL-18 facilitated CAR-mediated tumor clearance in the setting of low target antigen expression. Efficacy was further improved by fusing biologically active IL-18 to a leader peptide to direct release of constitutively active IL-18 via the secretory pathway [33]. Additionally, decoy-resistant versions of IL-18 have been generated by mutagenesis, obviating the natural antagonistic effects of IL-18BP on this cytokine [43, 47].

**TABLE 1:** Pre-clinical studies of IL-18-armored CAR and TCR-engineered T cells.

References	IL-18 format	Cell therapy	*In Vivo* cancer models	Mechanisms of action	Safety
Hu et al. [32]	Mouse and human Mostly constitutively active IL-18 but one experiment with NFAT-based inducible system	Human anti-mesothelin CAR T cells (4-1BB)	AsPC1 human pancreatic cancer in NSG mice	IL18R-dependent proliferation of CD4^+^ T cells induces CD8^+^ T cell expansion	Elevated serum IFN-γ, TNF-α, and IL-18. One C57BL/6 mouse died (potentially due to IL-18 toxicity)
		Human anti-CD19 CAR T cells (4-1BB)	Nalm6 human pre-B leukemia in NSG mice		
		Mouse anti-CD19 CAR T cells (4-1BB)	B16F10 mouse melanoma (CD19^+^) in C57BL/6 mice		
Avanzi et al. [33]	Mouse and human. Constitutively active IL-18. IL-2 leader peptide for improved secretion	Human anti-CD19 CAR T cells (CD28)	Nalm6 human pre-B leukemia in SCID/Beige mice	Autocrine activation of CAR T cells Activation of endogenous immune system with epitope spreading Re-polarization of macrophages DC activation and maturation and CD8^+^ T cell expansion	Elevated serum IL-6, TNF-α, IFN-γ and IL-18, but no safety concerns raised
		Mouse anti-CD19 CAR T cells (CD28)	EL4 mouse thymoma (human CD19^+^) in C57BL/6 mice		
		Mouse anti-MUC16ecto CAR T cells (4-1BB, CD28)	ID8 mouse ovarian carcinoma (human MUC16ecto^+^) in C57BL/6 mice		
Drakes et al. [34]	Mouse or human. Constitutively active IL-18	Mouse Pmel-1 T cells (CD8^+^ T cells from transgenic mice expressing TCR specific for gp100 peptide)	B16F10 mouse melanoma in C57BL/6 mice	Autocrine activation of CAR T cells CD8^+^ T cell expansion Reduced T cell exhaustion Fewer M2 macrophages Fewer MDSCs DC activation	Elevated serum IL-6, TNF-α, IFN-γ and IL-18, but no safety concerns raised
		Human NY-ESO-1 TCR-engineered T cells	A375 human melanoma in NSG mice		
Jaspers et al. [35]	Mouse Constitutively active IL-18	Mouse anti-DLL3 CAR T cells (4-1BB)	Mouse small cell lung cancer in C57BL/6 mice	CD8^+^ T cell expansion Less T cell exhaustion macrophage re-polarisation DC activation	Elevated serum IL-18 and IFN-γ, TNF-α Serum IL6 not elevated
		Human anti-DLL3 CAR T cells (CD28 or 4-1BB)	H82, H69 and SHP-77 human small cell lung cancer in NSG mice		
Ng et al. [36]	Human IL-18 Constitutively active Expressed in a separate vector	Human anti BCMA/TACI and anti-BAFF receptor dual CAR T cells (CD28, 4-1BB or both)	MOPC315.BM mouse plasmacytoma in BALB/c mice	Activation of host effector cells rather than direct T cell cytotoxicity Increased M1 macrophages Increased DCs	40% of mice treated with IL-18 armored 3rd generation CAR T-cells died Associated with elevated serum IL-6, GM-CSF, IL-10, IL-27, IFN-γ and IL-18
Chmielewski et al. [37]	Mouse and human IL-18 Expressed under the control of NFAT/IL2 minimal promoter In a separate vector to CAR.	Mouse anti-CEA CAR T cells (CD28)	Panc02 mouse pancreatic carcinoma (CEA+) in C57BL/6 mice	CD8^+^ T cells increase in effector phenotype (T-bet^+^, FoxO1^low^) Fewer immunosuppressive DCs, Tregs and M2 macrophages in the TME More NKG2D^+^ NK cells	Elevated serum IL-6, IL-27, and IL-18, but no change in IFN-γ, IL-2, GM-CSF or TNF-α
		Human anti-CEA CAR T cells (CD28)	A549 human lung cancer (CEA+) in Rag2−/− yc−/− mice		
Kunert et al. [38]	Mouse IL-18 Expressed under the control of NFAT/IL2 minimal promoter In a separate vector to CAR.	Mouse gp100/HLA-A2-specific TCR-engineered T cells	B16BL6 mouse melanoma (gp100^+^) in C57BL6/HLA-A2 mice	Enrichment of CD8^+^ T-cells in the TME No difference in myeloid populations	Elevated serum IL-18, but not IFN-γ, IL-2, IL-10, TNF-α or IL-18 Combination with IL-12 was toxic
Fisher-Riepe et al. [39]	Human IL-18 Expressed under the control of NFATsyn synthetic promoter and co-expressed with CAR using a single lentiviral vector	Human anti-GD2 CAR T cells (4-1BB)	CHLA-255 human neuroblastoma cells in NSG mice	CD8^+^ T cell expansion	CAR T cell-induced graft versus host disease was exacerbated by IL-18
Huang et al. [40]	Recombinant human or mouse IL-18 intraperitoneal injections (2 μg every 3 days)	Mouse anti-HER2 CAR T cells (4-1BB)	B16F10 (HER2^+^) mouse melanoma in C57BL/6 mice	*In vitro* IL-18-armored CAR T cells co-cultured with tumor cells downregulated pro-apoptotic genes and PD-1 and upregulated CCR12, CXC10 and IFN-γ *In vivo*, increased proportion of central memory T cells	No indication of toxicity Serum cytokines not measured
		Mouse OT-1 T cells (CD8^+^ murine T cells expressing ovalbumin-specific TCR)	G7-OVA mouse lymphoma in C57BL/6 mice		
		Human anti-HER2 CAR T cells (4-1BB)	SKOV3 human ovarian cancer and MCF-7 human breast cancer in NOD SCID mice		
Ma et al. [41]	Human or mouse IL-18 cDNA incorporated into a separate vector from CAR.	Human anti-GD2 CAR T cells (CD-28)	CHLA-255 human neuroblastoma cells in NSG mice	The focus of this study was IL-23 armoring	IL-18 armoring induced weight loss in CHLA-255 mouse model
		Mouse OT-1 T cells (CD8^+^ murine T cells expressing ovalbumin-specific TCR)	B16-OVA mouse melanoma in C57BL/6 mice		
Breman et al. [42]	Human IL-18 Constitutively active	NKG2D CAR T cells (NKG2D fused to CD3-ζ)	THP-1 human monocytic leukemia	​	Toxicity was observed and was abrogated using IL18-BP.
Olivera et al. [43]	Mouse 18BP-resistant IL-18 in combination with mouse IL-12 mRNA transiently introduced as mRNA via electroporation	Mouse Pmel-1 T Cells (CD8^+^ T cells from transgenic mice expressing TCR specific for gp100 peptide)	B16-OVA mouse melanoma in both flanks of C57BL/6 mice	The combination of IL-18 and IL-12 armoring increased expression of 2 O-glycans on T cells associated with advanced E-selectin adhesion and abscopal activity in non-injected tumors contralateral to injected tumors T cells also upregulated miR-155 which enhanced glucose metabolism and respiration	No indication of toxicity
		Mouse OT-1 T cells (CD8^+^ murine T cells expressing ovalbumin-specific TCR)			
		Mouse anti-gp75 CAR T cells (4-1BB)			
Ruixin et al. [44]	Mouse IL-18 Constitutively active	Mouse anti-EGFRvIII CAR T cells (CD28) with or without CXCR2	4T1 mouse mammary carcinoma in BALB/C mice	Reduced CAR T cell exhaustion	Elevated serum IL-6, IFN-γ, IL-10, IL-4 and TNF-α Safety improved in combination with CXCR2 (e.g., less elevation of IL-6 and IL-4)
			EO771 mouse mammary carcinoma in C57BL/6 mice		
Hull et al. [45]	Mouse and human; Granzyme B cleavable IL-18	Human parallel CAR T cells (anti-MUC1 CD28-containing CAR with T1E-targeted 4-1BB co-stimulatory receptor)	MDA-MB-468 human triple negative breast cancer in SCID Beige mice	Enhanced M1 macrophage polarization and increased DC frequency	Lethal toxicity in syngeneic models if IL-18 was constitutively active, but not seen with granzyme B format Toxicity was associated with elevated serum IL-6, MCP-1, GM-CSF, IFN-γ
		Mouse panErbB-specific CAR T cells (CD28)	B7E3 mouse head and neck squamous cell carcinoma in BALB/c mice		
Justicia-Lirio et al. [46]	Human IL-18 Doxycycline-inducible	Human anti-CD19 CAR T cells (4-1BB)	Namalwa (human Burkitt lymphoma model) in NSG mice	Proportion of T cells with memory phenotype increased	Only xenograft models so not much assessment of improved safety but it is the rationale
			MIA-PaCa2 (CD19^+^) human pancreatic adenocarcinoma in NSG mice		

All studies demonstrated improved anti-tumor activity linked to IL-18 armoring in at least one cancer model. Abbreviations: BAFF, B cell activating factor; CEA, carcinoembryonic antigen; CXCR2, CXC receptor 2; DLL3, delta-like ligand 3; EGFRVIII – VIII splice variant of epidermal growth factor receptor; GM-CSF, granulocyte macrophage colony-stimulating factor; IFN-γ, interferon γ; IL, interleukin; IL18R, interleukin 18 receptor; pmel, premelanosome protein; MCP1, monocyte chemoattractant protein 1; TACI, transmembrane activator and CAML interactor; TNF-α, tumor necrosis factor α; T1E – panErbB ligand, generated as a fusion protein derived from transforming growth factor α and epidermal growth factor.

In contrast, two studies reported that IL-18 armoring alone was not sufficient to boost CAR T cell anti-tumor activity. Ma et al. found that IL-18-armored anti-GD2 CAR T did not prolong the survival of mice engrafted with a CHLA-255 human neuroblastoma xenograft, instead inducing toxicity manifested as weight loss [41]. Such toxicity was also reported by Fisher-Riepe et al. in a similar model [39]. Additionally, Olivera et al. found that Pmel-1 TCR transgenic T cells and anti gp75 CAR T-cells achieved improved tumor control only if engineered to transiently co-express IL-12 and IL-18 mRNA, but not IL-18 alone [43]. This finding is in line with an earlier report that IL-12 upregulates IL-18Rβ and thus synergizes with IL-18 in inducing IFN-γ expression [48]. Contrasting with this however, Chmielewski et al. found that the combination of IL-12 and IL-18 did not improve anti-tumor activity beyond that observed with IL-18 alone, highlighting the importance of context in the biological actions of these cytokines [37]. The combination of IL-12 and IL-18 has also proven to be highly toxic in some studies [38].

## IL-18 has several mechanisms of anti-tumor action

The aforementioned pre-clinical studies have provided useful insights into mechanisms by which IL-18 can enhance tumor control. A key effect is autocrine stimulation via binding to T cell-associated IL-18R, amplifying the production of IFN-γ. In keeping with this, engineering of CAR T cells to produce membrane-bound IL-18 allowed engagement of IL-18R in *cis* and enhanced *in vitro* anti-tumor activity [49]. Single cell RNA sequencing analysis revealed that IL-18 armoring was linked to enhanced NF-κB signaling and gene expression associated with the cell cycle, T cell activation, interferon stimulation and antigen-presentation [36]. Further confirming the importance of autocrine stimulation, genetic knock out of IL-18R in IL-18 producing CAR or TCR-engineered T cells decreased efficacy in several models [32–34].

By binding IL-18R in *cis*, IL-18-armoring supports the proliferation of CD4^+^ CAR T cells, which, in turn, enabled the expansion of CD8^+^ CAR T cells [32]. Indeed, expansion of CD8^+^ T cells, both adoptively transferred and of host origin, has been a widely reported action of IL-18 by several groups [32–35, 38, 39]. Within the CD8^+^ T cell compartment, IL-18 has been reported to promote a CCR7^-^ effector memory and T-bet^+^ FoxO1^low^ terminal effector phenotype [32, 37]. In agreement, a recent clinical showed that most IL-18 armoring of anti-CD327 CAR T cells resulted in an amplification of CD8^+^ effector T cells [50]. Additionally, RNAseq data indicate that IL-18 armoring downregulates naïve T cell markers (CD27, CD127, CD62L) [36]. However, three other preclinical studies showed that IL-18 instead promoted the CD8^+^ CCR7^+^ CD62L^+^ central memory phenotype [33, 35, 46]. These discrepancies may be context-dependent, relating for example, to the specific CAR T cell and tumor model under study.

Although IL-18 drives T cell activation and differentiation, it has also been shown to decrease exhaustion [33, 35, 44], manifested also as a reduction in PD-1, TIM-3 and LAG-3 triple positive T cells [34]. Metabolic impact of IL-18 armoring was shown most convincingly in CAR-expressing γδ T cells, indicated by increased mitochondrial mass accompanied by upregulation of both the glucose transporter, GLUT1 and amino acid transporter, CD98 [45].

Importantly, effects of IL-18 armoring are not limited to autocrine actions. Avanzi et al. found that IL-18 secreted by CAR T cells also improved the anti-cancer activity of endogenous host T cells. Thus, IL-18-armored anti-CD19 CAR T cells increased the survival of mice engrafted with a mixture of CD19^+^ and CD19^NEG^ EL4 tumors. Splenocytes isolated from these mice that lacked CAR expression had an increased cytolytic and IFN-γ producing capacity when co-cultured with CD19^NEG^ tumors, unlike control splenocytes from mice treated with non-armored CAR T cells [33]. Nonetheless, when tested in the pmel-1 TCR transgenic model, impact on anti-tumor efficacy was less prominent if IL-18R expression was abrogated in host (rather than CAR T) cells only [34].

Interleukin 18 also has multiple effects on the myeloid compartment, albeit variable across different models. Several studies have reported macrophage re-polarization from an anti-inflammatory M2 (CD206^+^/MHC-II^lo^) to a pro-inflammatory M1 (MHCII^+^) phenotype [33–37, 45]. Additionally, the frequency of splenic and intra-tumoral DCs were increased, accompanied by a more mature and activated phenotype (CD11c^+^MHC-II^+^) [33–36]. Armoring with IL-18 has also been shown to reduce immunosuppressive intratumoral M2 macrophages alone [37], or in addition to both monocytic (CD11b^+^, Ly6C^+^) and granulocytic (CD11b^+^, Ly6G^+^) MDSCs [34]. Notably however, Kunert et al. found little impact of IL-18 on tumor-infiltrating myeloid cell numbers [38] while Jaspers et al. found that IL-18 upregulated PD-L1 on F4/80^+^ macrophages and DCs [35], an undesirable effect linked to IFN-γ production [51]. In line with this, combination therapy with IL-18-armored CAR T cells and a PD-L1 blocking antibody led to improved anti-tumor activity [35].

In addition to the autocrine and paracrine mechanisms described above, Huang et al. presented evidence that IL-18 may also act via an IL-18R independent mechanism [40]. Accordingly, when IL-18Rα was knocked out in both the host and the infused CAR T cells, recombinant IL-18 could still improve anti-tumor activity. Since IL-18Rβ cannot bind IL-18 with meaningful affinity alone, authors speculated that an additional unknown receptor may also be operative.

Finally, it should be noted that in some circumstances, dysregulated IL-18 activity has been linked with undesirable tumor-promoting effects [52–54]. However, this may reflect the actions of chronic low-level inflammation driven by this cytokine, contrasting with its effects when released acutely and at high-level by an IL-18 armored T cell.

## Toxicity associated with IL-18

Armoring with IL-18 is generally considered to be safer than with IL-12. Illustrating this, Drakes et al., showed that IL-12- but not IL-18-armored T cells caused fatal toxicity in sublethally irradiated mice [34]. Moreover, IL-12 has proven highly toxic in man when administered as a cytokine or in the context of armored TIL cells (Qi and Maher, manuscript under review), in contrast to the more modest side effects of IL-18 therapy [30, 31].

Nonetheless, there are a number of indicators to suggest that excessive IL-18 activity could also impose the risk of increased toxicity. First, several pre-clinical studies have demonstrated the ability of IL-18-armored T cells to induce severe and sometimes lethal toxicity [32, 36, 45]. Moreover, clinical evidence supports the important pro-inflammatory role of IL-18. Chronically elevated serum IL-18 is associated with pro-inflammatory diseases, such as hemophagocytic lymphohistiocytosis/macrophage activation syndrome (HLH/MAS) [55, 56]. These “IL-18opathies” are also characterised by CD8^+^ T cell expansion and macrophage hyperactivation [16, 57]. Driorio et al. found that patients experiencing CD19 CAR T cell-induced severe cytokine release syndrome (CRS) had a similar serum proteomic signature to that of HLH patients [58]. Evidence has also been presented that IFN-γ signaling promotes CRS [59]. Moreover, IL-18 has emerged as a biomarker associated with immune effector cell-associated neurotoxicity syndrome (ICANS) [58]. Consequently, by raising serum IFN-γ levels, it is logical that IL-18 armoring could potentially contribute increase risk and severity of both CRS and ICANS.

## Clinical experience with IL-18 armored T cells

Recently, the first clinical trial of IL-18-armored CD19 CAR T cells was reported in patients with B cell lymphoma (NCT04684563) [13]. Although 20 of 21 subjects had failed prior CAR T cell therapy, 11 achieved complete remission of disease by 3 months with a further 6 partial responses noted, giving a median duration of response of 9.6 months at median follow up of 17.5 months. Responses appeared to be more frequent if prior CAR T cell therapy had been with a CD28- rather than 4-1BB-containing product. These impressive data may also have been contributed to by the use of a shortened (3 days) manufacturing process, known to enhance T cell fitness. Authors presented evidence that active IL-18 was buffered effectively by IL-18BP in treated patients, mitigating risk of excessive toxicity. Cytokine release syndrome occurred in 13 subjects of which 3 reached grade 3 (correlated with higher CAR T-cell expansion), while neurotoxicity occurred in 3 patients (all grade 1–2) and there were no cases of hemophagocytic syndrome. While this would generally be considered an acceptable safety profile, it should be noted however that one subject developed tocilizumab/corticosteroid-refractory CRS and was ultimately treated with IL-18 binding protein. Elsewhere it is reported that one (perhaps the same) subject developed transient pulmonary edema in the context of grade 3 CRS, which was deemed a dose-limiting toxicity (DLT) that required expansion of the 3 × 10^7^ cell dose level to 6 subjects.

In a second clinical trial, 5 acute myeloid leukemia patients were treated with IL-18-armored anti-CD371 CAR T cells (NCT06017258) [50]. Three achieved minimal residual disease negative disease status, also confirming the therapeutic activity of this experimental approach. All 5 treated patients developed CRS. Onset of symptoms correlated with a peak in serum IL-18 and IFN-γ levels, as well as NK cell expansion and activation suggesting that biologically active IL-18 was present. Both patients who received the highest planned dose of 3 × 10^5^ cells/kg experienced DLTs, namely, prolonged cytopenias and grade 4 CRS respectively. The latter DLT was resistant to two doses of tocilizumab and ultimately was successfully treated with the IFN-γ-blocking antibody, emapalumab. In short, these clinical data provide strong clinical support for the ability of IL-18 CAR armoring to boost efficacy, but highlight the fact that this approach may accentuate the risk of inflammatory toxicity in some cases.

## Strategies to mitigate IL-18 mediated toxicity

Given the aforementioned considerations, efforts have been made to restrict the functional impact of IL-18 armoring systems to the TME. Conceptually, this is particularly appropriate for solid tumors given their propensity to originate from and metastasize to parenchymal organs - meaning that effective technologies would minimize unwanted IL-18 activity in the circulation. One commonly used system entails placing the IL-18 cDNA under the transcriptional control of a Nuclear Factor of Activated T cells (NFAT)-based promoter. Since NFAT upregulation is coupled to activation of CAR by its target antigen, IL-18 is preferentially produced in the TME [60, 61]. Chmielewski et al. demonstrated the safety and efficacy of T cells armored with IL-18 under the control of NFAT/IL2 minimal promoter [37]. Using this dual vector approach, they observed no toxicity and no increase in serum IFN-γ. However, serum levels of IL-18, IL-6 and IL-27 were elevated indicating that this system may not have been completely stringent. In keeping with this, NFAT-regulated IL12 constructs proved lethal in mice [62] and also caused toxicity in clinical trials [63], likely due to non-specific upregulation of NFAT by signals not related to binding of target antigen. Providing reassurance, the same system was used to express IL-18 in TCR-engineered T cells without evident toxicity or detectable levels of circulating IFN-γ or tumor necrosis factor (TNF) α [38]. This suggests that a degree of leakiness of the system may be tolerable for IL-18 armoring in light of the lower toxicity seen with this cytokine compared to IL-12. More recently, a similar NFAT plus synthetic TATA box regulated IL-18 expression system has been incorporated into a single lentiviral vector system together with a GD2 specific CAR [39]. When tested in preclinical xenograft model of GD2-expressing malignancy, superior anti-tumor efficacy was once again demonstrated. This system has now been advanced to early phase clinical testing in patients with GD2-expressing malignancies (EU CT 2022– 501725–21–00). Finally, Hu et al. have also independently described an NFAT-regulated IL-18 armoring technology as a device to improve the safety of this approach [32].

As an alternative approach, Justicia-Lirio et al. developed a doxycycline-inducible IL-18 technology [46]. Anti-CD19 CAR T cells armored with this system showed excellent safety and efficacy in xenograft mouse models. This strategy was also used to improve safety of IL-12 armoring, providing a testament to its stringency [62].

A distinct strategy to restrict IL-18 activity to the TME was developed by Hull et al. [45] Since T cells lack caspase 1 activity, the caspase 1 proteolytic cleavage site in pro-IL18 was modified to one favored by granzyme B (GzB-IL18). The resulting GzB-IL18 propeptide is constitutively released in an inactive state by the armored CAR T cells. However, it selectively acquires biological activity when CAR T cell degranulation occurs, owing to co-localization with released granzyme B. In a syngeneic mouse model of head and neck squamous cell carcinoma, mice treated with Gzb-IL18-armored panErbB-specific CAR T cells had an execllent safety profile and improved survival due to enhanced tumor control. In contrast, armoring of panErbB CAR T cells with constitutively active IL-18 was lethal in this model. Toxicity was associated with increased serum levels of several cytokines, including IFN-γ.

## Discussion

IL-18 armoring improves the efficacy of CAR and TCR-engineered T cell therapies, as shown in numerous pre-clinical studies. Moreover, recent clinical experience supports the utility of this approach in hematological malignancies. However, these studies also suggest the potential for uncontrolled IL-18 activity to aggravate CAR T cell-mediated toxicities such as CRS and ICANs, especially if coupled to a CAR that already has significant toxic potential. This provides a strong rationale for the use of engineering strategies that can improve safety of IL-18 armoring, while preserving or even improving its efficacy. Three such strategies have been discussed here, namely,: NFAT- or doxycycline-controlled IL-18 transcription or modification of the cleavage site within pro-IL-18 to one favored by granzyme B. The first of these technologies, NFAT-inducible IL-18, is already undergoing clinical evaluation. Interleukin 18-armored CAR T cells are currently being tested in a small number of clinical trials (Table 2). Ultimately, only the results of these and additional studies will convincingly show whether IL-18 significantly contributes to anti-tumor efficacy without causing toxicities beyond tolerable limit. In the meantime, additional pre-clinical studies will provide further insights into the various mechanisms by which secreted IL-18 boosts the anti-tumor activity of both engineered T cells and the endogenous immune system.

**TABLE 2:** Ongoing clinical trials of IL-18-armored CAR T cells. Search conducted on https://clinicaltrials.gov, and https://euclinicaltrials.eu, both accessed 17th December 2025.

Trial identificator	Sponsor	Name	Status
NCT04684563 [13]	University of Pennsylvania	Phase I trial of huCART19-IL18 cells in patients with relapsed or refractory CD19^+^ cancers	Active, not recruiting
NCT05989204	University of Pennsylvania	TmCD19-IL18 in CD19^+^ cancers	Recruiting
NCT06017258 [50]	Memorial Sloan Kettering Cancer Center	A study of CD371-YSNVZIL-18 CAR T cells in people with acute myeloid leukemia	Recruiting
NCT06287528	Memorial Sloan Kettering Cancer Center	A study of 19-28z/IL-18 in People with acute Lymphoblastic Leukemia (ALL)	Recruiting
NCT05783570	Eutilex	To evaluate the safety, tolerability and preliminary efficacy of EU307	Recruiting
EU CT 2022– 501725–21–00	Muenster University	A phase I safety, dose finding and feasibility trial of GD2IL18CART in patients with relapsed or refractory GD2 positive solid cancers	Recruiting
